# Supplementary material for: Structural characterization and dynamics of AdhE ultrastructures from Clostridium thermocellum show a containment strategy for toxic intermediates
Source: eLife. 2025 Jun 27;13:RP96966. doi: 10.7554/eLife.96966 (PMC12204686; doi:10.7554/eLife.96966)
Supplement: Supplementary file 1. — (A). Sequences of AdhE primers. Nucleotide sequences of the primers used in this study. (B). Sequences of the AdhE constructs. Amino acid sequences of the two proteins, E. coli and C. thermocellum AdhE, used in this study. (C). Data collection and processing. The statistics describing the cryo-electron microscopy (cryo-EM) data processing performed in this study. [file elife-96966-supp1.docx]

**Supplementary File 1A: Primers, plasmids and strains used in this study**

| **Strain** | **Plasmid** | **Target gene** | **Genetic tag** | **Primers used for cloning** | |
| --- | --- | --- | --- | --- | --- |
|  |  |  |  | **Name** | **Sequence** |
| M7-2 | pCB16-1 | E. coli AdhE | N-histag | 1184 | tgagatccggctgctaacaaa |
|  |  |  |  | 1252 | ccggatatagttcctcctttcag |
|  |  |  |  | 1524 | CATGGTATATCTCCTTCTTAAAGTTAAACAA |
|  |  |  |  | 1710 | taattttgtttaactttaagaaggagatataccatgcaccaccaccaccaccacgagaacctgtatttccagggtatgGCTGTTACTAATGTCGCTGA |
|  |  |  |  | 1711 | ctcagcttcctttcgggctttgttagcagccggatctcaAGCGGATTTTTTCGCTTTTTTCTC |
|  |  |  |  | 1712 | CGTTCCGACCACTAACCCGAC |
|  |  |  |  | 1713 | GCTGAACTGGCAGGCTTCTCT |
|  |  |  |  | 1714 | TTCGAAGTAGAAGCGGACCCG |
| M8-2 | pCB17-12 | C. thermocellum AdhE | N-histag | 1184 | tgagatccggctgctaacaaa |
|  |  |  |  | 1252 | ccggatatagttcctcctttcag |
|  |  |  |  | 1524 | CATGGTATATCTCCTTCTTAAAGTTAAACAA |
|  |  |  |  | 1715 | ttttgtttaactttaagaaggagatataccatgcaccaccaccaccaccacgagaacctgtatttccagggtatgACGAAAATAGCGAATAAATACGAAG |
|  |  |  |  | 1716 | ctcagcttcctttcgggctttgttagcagccggatctcaTTTCTTCGCACCTCCGTAATA |
|  |  |  |  | 1717 | GTTGTGCCCGGTACTGGCAAT |
|  |  |  |  | 1571 | TTATAAGCCCACACCCCAGGG |
|  |  |  |  | 1572 | GTAGCTGACGGTGGATTTGGCC |
|  |  |  |  | 1573 | GACACCATAATTGCGGTCGGC |

Supplementary File 1B: Protein sequences used in this study

| Construct name | Sequence |
| --- | --- |
| **E. coli AdhE in pCB16-1** | MHHHHHHENLYFQGMAVTNVAELNALVERVKKAQREYASFTQEQVDKIFRAAALAAADARIPLAKMAVAESGMGIVEDKVIKNHFASEYIYNAYKDEKTCGVLSEDDTFGTITIAEPIGIICGIVPTTNPTSTAIFKSLISLKTRNAIIFSPHPRAKDATNKAADIVLQAAIAAGAPKDLIGWIDQPSVELSNALMHHPDINLILATGGPGMVKAAYSSGKPAIGVGAGNTPVVIDETADIKRAVASVLMSKTFDNGVICASEQSVVVVDSVYDAVRERFATHGGYLLQGKELKAVQDVILKNGALNAAIVGQPAYKIAELAGFSVPENTKILIGEVTVVDESEPFAHEKLSPTLAMYRAKDFEDAVEKAEKLVAMGGIGHTSCLYTDQDNQPARVSYFGQKMKTARILINTPASQGGIGDLYNFKLAPSLTLGCGSWGGNSISENVGPKHLINKKTVAKRAENMLWHKLPKSIYFRRGSLPIALDEVITDGHKRALIVTDRFLFNNGYADQITSVLKAAGVETEVFFEVEADPTLSIVRKGAELANSFKPDVIIALGGGSPMDAAKIMWVMYEHPETHFEELALRFMDIRKRIYKFPKMGVKAKMIAVTTTSGTGSEVTPFAVVTDDATGQKYPLADYALTPDMAIVDANLVMDMPKSLCAFGGLDAVTHAMEAYVSVLASEFSDGQALQALKLLKEYLPASYHEGSKNPVARERVHSAATIAGIAFANAFLGVCHSMAHKLGSQFHIPHGLANALLICNVIRYNANDNPTKQTAFSQYDRPQARRRYAEIADHLGLSAPGDRTAAKIEKLLAWLETLKAELGIPKSIREAGVQEADFLANVDKLSEDAFDDQCTGANPRYPLISELKQILLDTYYGRDYVEGETAAKKEAAPAKAEKKAKKSA- |
| **C. thermocellum AdhE in pCB17-12** | MHHHHHHENLYFQGMTKIANKYEVIDNVEKLEKALKRLREAQSVYATYTQEQVDKIFFEAAMAANKMRIPLAKMAVEETGMGVVEDKVIKNHYASEYIYNAYKNTKTCGVIEEDPAFGIKKIAEPLGVIAAVIPTTNPTSTAIFKTLIALKTRNAIIISPHPRAKNSTIEAAKIVLEAAVKAGAPEGIIGWIDVPSLELTNLVMREADVILATGGPGLVKAAYSSGKPAIGVGAGNTPAIIDDSADIVLAVNSIIHSKTFDNGMICASEQSVIVLDGVYKEVKKEFEKRGCYFLNEDETEKVRKTIIINGALNAKIVGQKAHTIANLAGFEVPETTKILIGEVTSVDISEEFAHEKLCPVLAMYRAKDFDDALDKAERLVADGGFGHTSSLYIDTVTQKEKLQKFSERMKTCRILVNTPSSQGGIGDLYNFKLAPSLTLGCGSWGGNSVSDNVGVKHLLNIKTVAERRENMLWFRTPEKIYIKRGCLPVALDELKNVMGKKKAFIVTDNFLYNNGYTKPITDKLDEMGIVHKTFFDVSPDPSLASAKAGAAEMLAFQPDTIIAVGGGSAMDAAKIMWVMYEHPEVDFMDMAMRFMDIRKRVYTFPKMGQKAYFIAIPTSAGTGSEVTPFAVITDEKTGIKYPLADYELLPDMAIVDADMMMNAPKGLTAASGIDALTHALEAYVSMLATDYTDSLALRAIKMIFEYLPRAYENGASDPVAREKMANAATIAGMAFANAFLGVCHSMAHKLGAFYHLPHGVANALMINEVIRFNSSEAPTKMGTFPQYDHPRTLERYAEIADYIGLKGKNNEEKVENLIKAIDELKEKVGIRKTIKDYDIDEKEFLDRLDEMVEQAFDDQCTGTNPRYPLMNEIRQMYLNAYYGGAKK- |

| **Supplementary File 1C: Data collection and processing.** | | | |
| --- | --- | --- | --- |
|  | **Extended Final** | **Extended Model Building** | **Compact Model Building** |
| **Data collection** |  |  |  |
| Microscope/camera | Krios/Falcon 4 | Talos Arctica/K3 | Krios/Falcon 4 |
| Voltage (kV) | 300 | 200 | 300 |
| Magnification | 96,000 | 45,000 | 96,000 |
| Electron dose (e^-^/Å^2^) | 60 | 90 | 60 |
| Pixel Size (Å) | 0.82 | 0.89 | 0.82 |
| Defocus Range (μm) | 1.5-3.0 | 1.2-2.5 | 1.0-3.0 |
| Processing | Single particle analysis | Single particle analysis | Single particle analysis |
| Symmetry imposed | C1 | C1 | C1 |
| Initial particle images (no.) | 504,494 | 352,061 | 959,561 |
| Final particle images (no.) | 90,912 | 103,973 | 64,977 |
| Map resolution (Å)-(FSC threshold model) | 3.28-(0.143) | 3.8-(0.143) | 3.93-(0.143) |
| **Refinement and validation** |  |  |  |
| Map sharpening (B-factor) (Å^-2^) | -97.02 | -99.00 |  |
| **Model composition** |  |  |  |
| No. of chains | 11 | 10 |  |
| Atmos (no.) | 30,785 | 31,285 |  |
| Residues (no.) | 3,994 | 4,047 |  |
| Ligands (no.) | Fe^2+^ (5) | Fe^2+^ (4) |  |
| Bond lengths (Å) | 0.003 | 0.004 |  |
| Bond angles (˚) | 0.590 | 0.633 |  |
| Ramachandran favored % | 95.26 | 93.92 |  |
| Ramachandran allowed % | 4.74 | 6.01 |  |
| Ramachandran outliers % | 0.00 | 0.08 |  |
| Rotamers outliers % | 0.00 | 0.00 |  |
| MolProbity score | 1.65 | 1.92 |  |
| Clashscore | 5.84 | 9.80 |  |
| CC (mask) | 0.86 | 0.79 |  |
| CC (box) | 0.70 | 0.70 |  |
| CC (peaks) | 0.70 | 0.62 |  |
| CC (volume) | 0.82 | 0.78 |  |
| Mean CC for ligands | 0.84 | 0.77 |  |
